# WHIRLY proteins maintain seed longevity by effects on seed oxygen signalling during imbibition

**DOI:** 10.1042/BCJ20230008

**Published:** 2023-07-06

**Authors:** Rachel E. Taylor, Wanda Waterworth, Christopher E West, Christine H. Foyer

**Affiliations:** 1 The Centre for Plant Sciences, Faculty of Biological Sciences, University of Leeds, Leeds LS2 9JT, U.K.; 2 School of Biosciences, College of Life and Environmental Sciences, University of Birmingham, Edgbaston B15 2TT, U.K.

**Keywords:** ageing, climate change, germination, heat shock proteins, longevity, seeds

## Abstract

The WHIRLY (WHY) family of DNA/RNA binding proteins fulfil multiple but poorly characterised functions in plants. We analysed WHY protein functions in the Arabidopsis *Atwhy1*, *Atwhy3*, *Atwhy1why3* single and double mutants and wild type controls. The *Atwhy3* and *Atwhy1why3* double mutants showed a significant delay in flowering, having more siliques per plant but with fewer seeds per silique than the wild type. While germination was similar in the unaged high-quality seeds of all lines, significant decreases in vigour and viability were observed in the aged mutant seeds compared with the wild type. Imbibition of unaged high-quality seeds was characterised by large increases in transcripts that encode proteins involved in oxygen sensing and responses to hypoxia. Seed aging resulted in a disruption of the imbibition-induced transcriptome profile such that transcripts encoding RNA metabolism and processing became the most abundant components of the imbibition signature. The imbibition-related profile of the *Atwhy1why3* mutant seeds, was characterised by decreased expression of hypoxia-related and oxygen metabolism genes even in the absence of aging. Seed aging further decreased the abundance of hypoxia-related and oxygen metabolism transcripts relative to the wild type. These findings suggest that the WHY1 and WHY3 proteins regulate the imbibition-induced responses to oxygen availability and hypoxia. Loss of WHY1 and WHY3 functions decreases the ability of Arabidopsis seeds to resist the adverse effects of seed aging.

## Introduction

Seeds and seed germination are the foundation of agriculture [1] and they are also of paramount importance to food security and the conservation of wild species. Considerable economic losses result from sub-optimal seed performance, undermining food security and livelihoods. Climate change is predicted to increase the frequency of heatwaves and droughts, factors that will adversely affect seed variability, germination and plant establishment and therefore further exacerbate economic losses and decrease the predictability of seed yield and quality for the farmer.

Germination occurs when the radicle emerges from the seed coat following imbibition (rehydration via water uptake). The time taken for a seed lot to germinate after imbibition is defined by vigour, whereas the final fraction of germinated seeds is determined by the percentage viability [2]. A component of successful germination is the maintenance of the reduction/oxidation (redox) ability of the cells and the repair of DNA damage accumulated during seed development process, drying during maturation, and germination. The rehydration process requires substantial metabolic rewiring leading to nucleic acid synthesis, initiation of DNA repair and activation of antioxidant mechanisms. DNA damage accumulates during seed storage and DNA repair is important during the initial rehydration process to promote the successful emergence of the radicle [2].

Seed viability and vigour are multigenic traits [3]. The performance of seeds and subsequent plant development are important to agroindustry and natural ecosystems. Some seeds undergo a period of dormancy, during which they are programmed not to germinate until a dormancy-breaking signal is received. Freshly harvested seeds generally have a relatively high dormancy level that is gradually released during dry seed storage. Seed dormancy ends when appropriate environmental cues including temperature, light and phytohormones, such as gibberellic acid (GA) and abscisic acid (ABA), are integrated by a spatially embedded decision-making centre to establish seed germination [4,5].

While the genetic determinants of viability and vigour have not yet been fully elucidated, reduced seed quality is often associated with damage to cellular structures including genomic DNA [2]. The WHIRLY (WHY) family of single stranded (ss)DNA-binding proteins plays an important role in the recognition, stabilisation, and processing of ssDNA during DNA replication, recombination and telomere maintenance during plant growth and development and have functions in the maintenance of nuclear and organellar genomes [7]. For example, WHY1 and WHY3 maintain the plastid genome and act as positive regulators of RNA/DNA hybrids through the recruitment of plastid-encoded RNA polymerases [6]. The *Atwhy1why3* double mutants accumulate rearranged chloroplast DNA through microhomology-mediated break-induced replication [7]. In addition, WHY proteins act as transcription factors in the nucleus that regulate plant development and abiotic and biotic stress responses [8,9].

The WHY1 and WHY3 proteins are localised to plastids and the nucleus, while the WHY2 protein is targeted to mitochondria but may also traffic to plastids and the nucleus. WHY regulates DNA replication in plastids, with functions in DNA repair and the maintenance of plastid genome stability [10–12]. In the nucleus, WHY1 is required for telomere maintenance [13] and together with WHY3, bind to the promoters of senescence associated and defence genes, such as WRKY53 transcription factor [11,14–17]. The presence of WHY1 on the WRKY53 promoter prevents the enrichment of H3K4me3 and H3K9ac. This favours recruitment of RNA polymerase II to the preinitiation complex. WHY1 inhibits H3K4me3 enrichment while promoting H3K9ac accumulation and RNA polymerase II recruitment, and so regulates WRKY53 expression [18].

Seeds of the Arabidopsis *Atwhy1* mutants and seeds of transgenic lines expressing WHY1 in the nucleus were reported to be insensitive to the phytohormones ABA and salicylic acid (SA) during germination [19]. In contrast, the seeds of transgenic lines overexpressing *AtWHY1* (in both plastids and nuclei) were hypersensitive to ABA [19]. These findings suggest that WHY protein influence the phytohormone-dependent regulation of seed germination [20]. Overexpression of the potato WHY2 (SlWHY2) protein in *Nicotiana benthamiana* enhanced drought stress tolerance during seed germination and seedling growth [21]. Moreover, seed viability was decreased in the *Atwhy2* mutants, which exhibit an altered mitochondrial structure, with disordered nucleoids and increased expression of *AtWHY3* transcripts compared with the wild type [22].

WHY proteins are involved in the regulation of plant development as well as abiotic and biotic stress responses [8], but relatively little is known about their roles in seeds. The following experiments were therefore performed to determine whether WHY proteins are important in protection against seed aging. The data presented here demonstrate that the WHY1 and WHY3 proteins play important roles in germination and seed longevity. The transcriptome profiles of both the wild type and *Atwhy1why3* double mutant seeds was disrupted by aging. The imbibition-related profile of the *Atwhy1why3* mutant seeds was characterised by decreased expression of hypoxia-related and oxygen sensing genes even in the absence of aging. Seed aging further decreased the abundance of hypoxia-related and oxygen metabolism transcripts relative to the wild type. Taken together, these data demonstrate that WHY1 and WHY3 fulfil a key role in seed longevity and germination and serve to identify the mechanisms involved in aging hypersensitivity.

## Results

**Figure 1.**
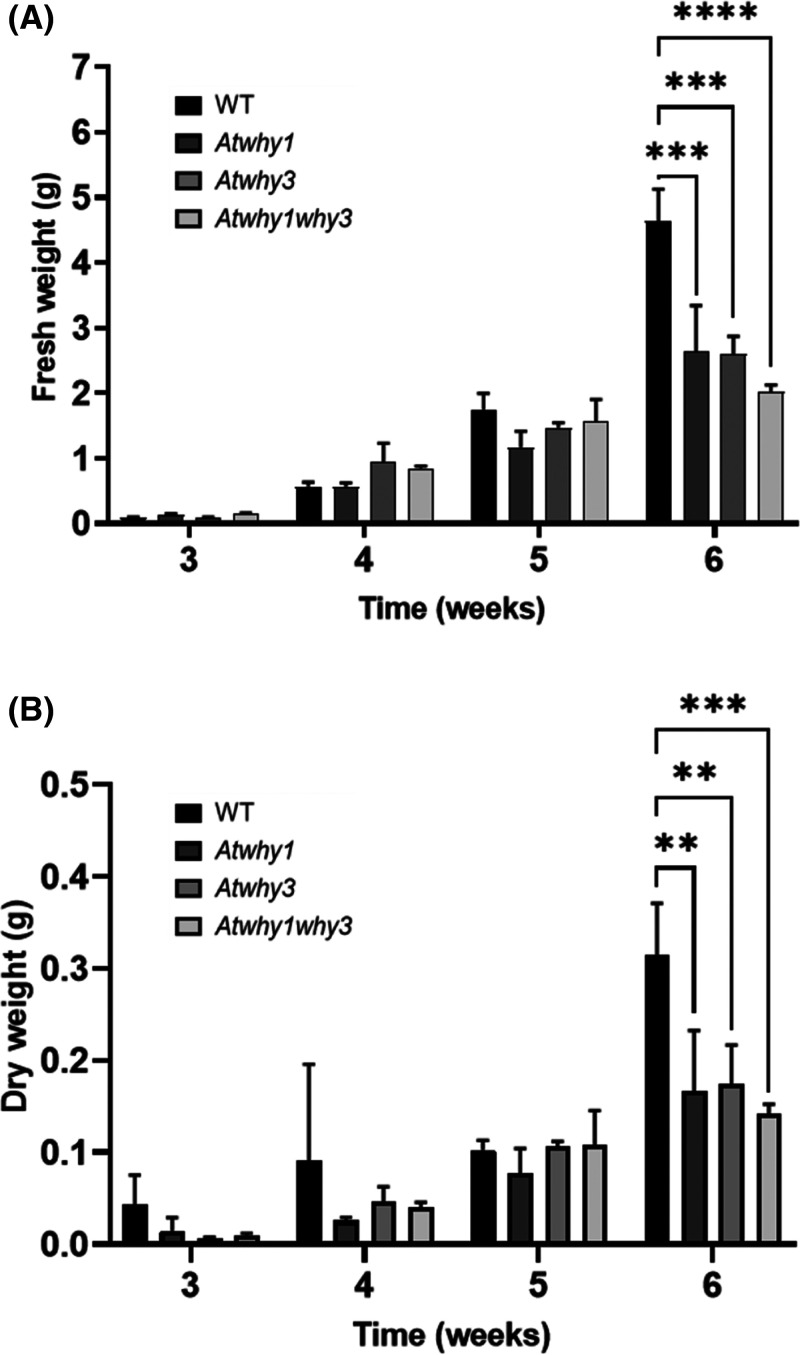
Rosette biomass accumulation in wild type *Arabidopsis thaliana* and in mutants lacking the WHIRLY 1 and/or 3 proteins. A comparison of the fresh (**a**) and dry (**b**) weight of the wild type and the *Atwhy1*, *Atwhy3* and *Atwhy1why3* mutants from 3–6 weeks after cold stratification. Means were calculated from three biological replicates. Error bars were standard error of means and students *t*-tests were conducted to compare each mutant to the WILD TYPE at each time point: ** *P* < 0.01; *** *P* < 0.001; **** *P* < 0.0001.

**Figure 2.**
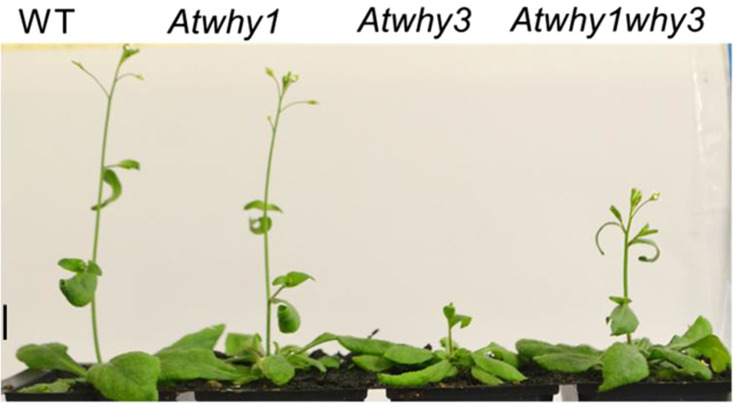
Representative images of the wild type (WILD TYPE) and the *Atwhy1*, *Atwhy3* and *Atwhy1why3* mutants at 6 weeks post germination. Scale bar was 10 mm.

**Figure 3.**
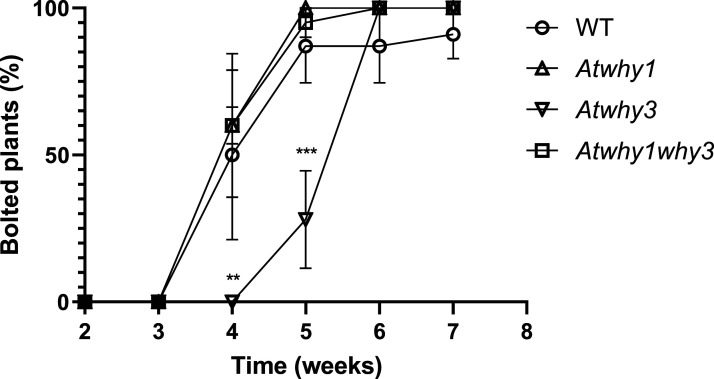
A comparison of the numbers (%) of wild type (WILD TYPE) of plants and *Atwhy1*, *Atwhy3* and *Atwhy1why3* mutants that had bolted at 6 weeks. Means were calculated from four biological replicates. Error bars were standard error of means and a two-way ANOVA was conducted to compare mutants within the same time point, *P*-values shown are compared with the WILD TYPE at each time point: ** *P* < 0.01; *** *P* < 0.001.

**Figure 4.**
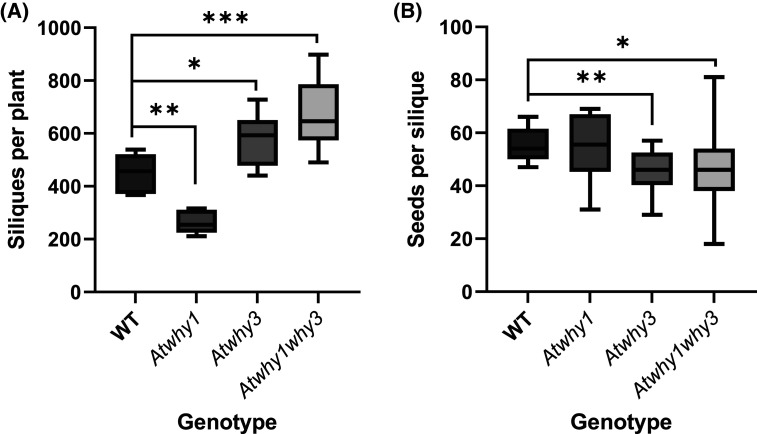
Silique numbers and seed numbers per silique in wild type *Arabidopsis thaliana* and in mutants lacking the WHIRLY 1 and/or 3 proteins. A comparison of the numbers of siliques per plant (**a**) and number of seeds per silique (**b**) in the wild type (WILD TYPE) and the *Atwhy1*, *Atwhy3* and *Atwhy1why3* mutants. Means were calculated from four siliques per plant and three plants per genotype. Error bars were standard error of means and students *t*-tests were conducted to compare each mutant to the WILD TYPE at each time point: * *P* < 0.05; ** *P* < 0.01; *** *P* < 0.001.

Whirly mutant lines were compared with Col-0 controls to determine any effects of the mutations on plant growth. The early growth and development of the *Atwhy*1, *Atwhy3* and *Atwhy1why3* mutants was similar to that of the wild type. However, after 6 weeks, the mutant lines had accumulated significantly less rosette biomass than the wild type (Figure 1). Moreover, the appearance of the flowering stem was delayed in the *Atwhy3* mutants relative to the other lines (Figures 2 and 3). Silique and seed production were also altered on the *Atwhy1*, *Atwhy3* and *Atwhy1why3* mutants relative to the wild type controls (Figure 4). In particular, the *why1* mutants had fewer siliques per plant than the wild type plants (Figure 4a). In contrast, the *Atwhy3* and *Atwhy1why3* mutants had significantly more siliques per plant than the wild type (Figure 4a). However, the *Atwhy3* and *Atwhy1why3* mutants had lower seed numbers per silique than the wild type (Figure 4b). We did not analyse whether the lower seed numbers were caused by a reduction in ovule number, failure of ovules to be fertilised or ovule abortion.

### Effects of seed aging on germination

**Figure 5.**
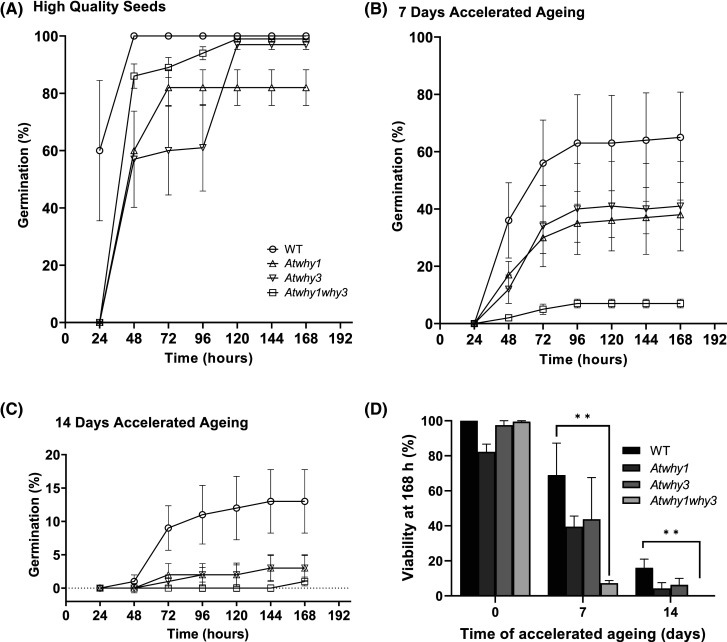
A comparison of germination properties in the wild type (WILD TYPE) and the *Atwhy1*, *Atwhy3* and *Atwhy1why3* mutants immediately after cold stratification. The germination of (**a**) high-quality aged seeds, (**b**) seeds that have undergone 7 days of accelerated aging or (**c**)14 days of accelerated aging . Error bars are standard error of means calculated from three replicates. (**d**) Viability of the three *Atwhy* mutant seeds compared with the WILD TYPE at 7 days after cold stratification after different accelerated aging treatments. Each treatment is shown per genotype. Error bars are standard error of means calculated from three replicates. *T*-test against WILD TYPE per aged section ** *P* < 0.01.

Seeds of each genotype were exposed to accelerated aging conditions involving elevated temperature (35°C) and relative humidity (83%) for 7 or 14 days. Germination was then compared in the aged and unaged seed batches. Seed viability was measured 7 days (168 h) post cold stratification (Figure 5d). The high-quality unaged seeds of all genotypes maintained a high vigour at this time point (wild type, 100%; *Atwhy1*, 82.3%; *Atwhy3*, 97.4%; *Atwhy1why3*, 99.6%). There were no significant differences between the mutants and the wild type in the absence of accelerated aging (Figure 5d). Aged seeds showed a marked delay in germination compared with unaged controls (Figure 5a–c). Germination viability was reduced in the 7 day-aged seeds of all genotypes, the effect being most pronounced in the mutant lines (wild type, 65.7%; *Atwhy1*, 38.7%; *Atwhy3*, 47.14%; *Atwhy1why3*, 7.38%). Germination viability was reduced further in the seeds aged for 14 days (wild type, 13.2%; *Atwhy1*, 3.14%; *Atwhy3*, 3.89%; *Atwhy1why3*, 1%). The decrease in seed viability was greater in the aged mutant seeds than the wild type (Figure 5b,c). Significant decreases in the viability of *Atwhy1why3* double mutant seeds was observed after 7 (*P* = 0.014) and 14 days of aging (*P* = 0.0498) compared with the aged wild type seeds (Figure 5b,c). The mean germination time (germination vigour) was decreased only in the *Atwhy1why3* double mutant after 14 days of aging (Supplementary Figure S1).

### RNA-Seq analysis

Transcriptome profiling analysis was performed on unaged and aged seeds of the wild type and *Atwhy1why3* double mutant seeds in order to compare the molecular basis for aging hypersensitivity in both genotypes. RNA was extracted from seeds that had been subjected to the following treatments: (1) dry (0 h imbibition); (2) imbibed (6 h) and (3) aged (7 day accelerated aging and 6 h imbibition). Changes in the transcript profiles of the different seed samples was determined using principal component analysis (Supplementary Table S1; Supplementary Figure S2). The variation between the samples of aged seeds was greater than that found in high-quality seeds of both genotypes (Supplementary Figure S2).

### Imbibition-induced changes in the transcript profiles of non-aged and aged wild type seeds

**Figure 6.**
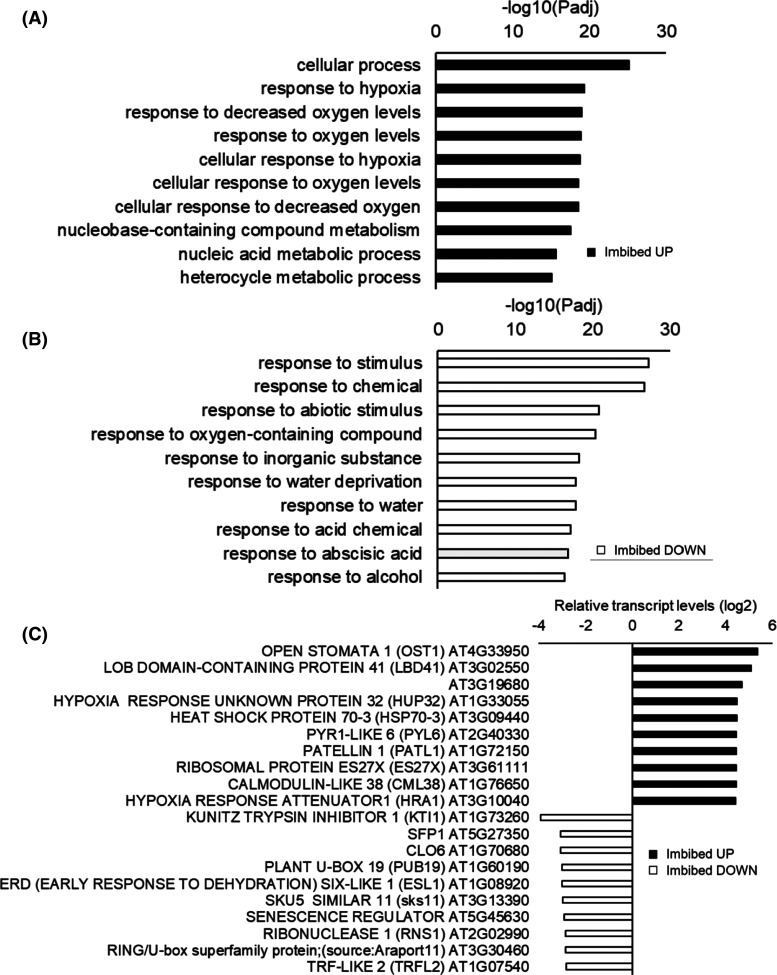
A comparison of the transcript profiles of imbibed unaged seeds compared with dry seeds. Gene ontology (GO) enrichment after 6 h imbibition relative to dry seeds (**a**) and fold change in expression of the most differentially expressed genes (DEGs) following imbibition of high-quality seeds (**b**).

The transcript profiles of the imbibed non-aged seeds showed a strong signature of hypoxia and oxygen sensing related genes compared with the dry controls (Figure 6a). Transcripts encoding components involved in nucleic acid metabolism were also increased in abundance at 6 h of imbibition (Figure 6a). Conversely, transcripts encoding proteins related to drought, ABA and alcohol were decreased following imbibition in unaged seeds (Figure 6a). Transcripts related to seed protein metabolism, as well as heat shock proteins (HSPs), were highly expressed following imbibition in wild type seeds compared with the dry controls (Figure 6b). Conversely, transcripts encoding Kunitz trypsin inhibitor 1 (KTI1) and early response to dehydration 1 (ESL1) were lower in imbibed wild type seeds compared with the dry controls (Figure 6b). Several hypoxia-related genes appear in the list of genes with the greatest level of differentially expression upon imbibition of high-quality seeds (Figure 6c).

**Figure 7.**
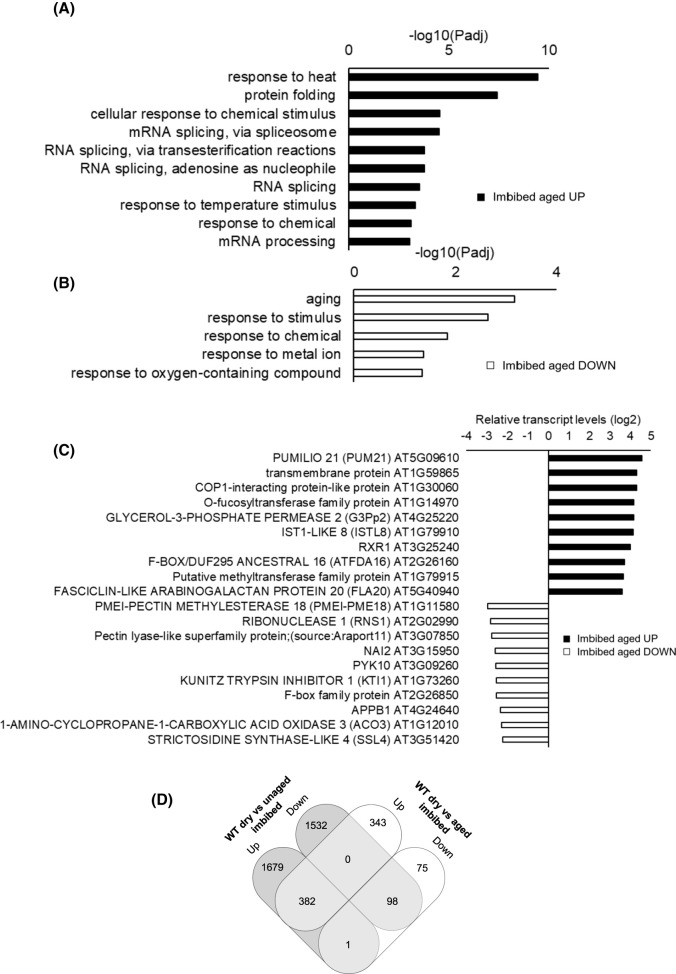
A comparison of the transcript profiles of imbibed aged seeds relative to dry controls. Gene ontology (GO) enrichment in 6 h imbibed aged seeds relative to dry seeds (**a**), fold change in the expression of differentially expressed genes (DEGs) following imbibition of aged seeds (**b**) and Venn diagram comparisons of the numbers of DEGs in common or specific to each treatment (**c**).

The responses of gene expression to imbibition were very different in the high-quality seed to that observed in the aged seeds (Figure 7). The major GO terms showing increased expression following imbibition of aged seeds were related to heat shock and protein folding, RNA slicing and folding (Figure 7a,b). There was little overlap in the patterns of gene expression observed in response to imbibition in the aged and unaged wild type seeds (Figure 7c).

### Comparisons of the imbibition-induced changes transcript profiles of wild type and *Atwhy1why3* mutant seeds

#### Imbibed unaged seeds

**Figure 8.**
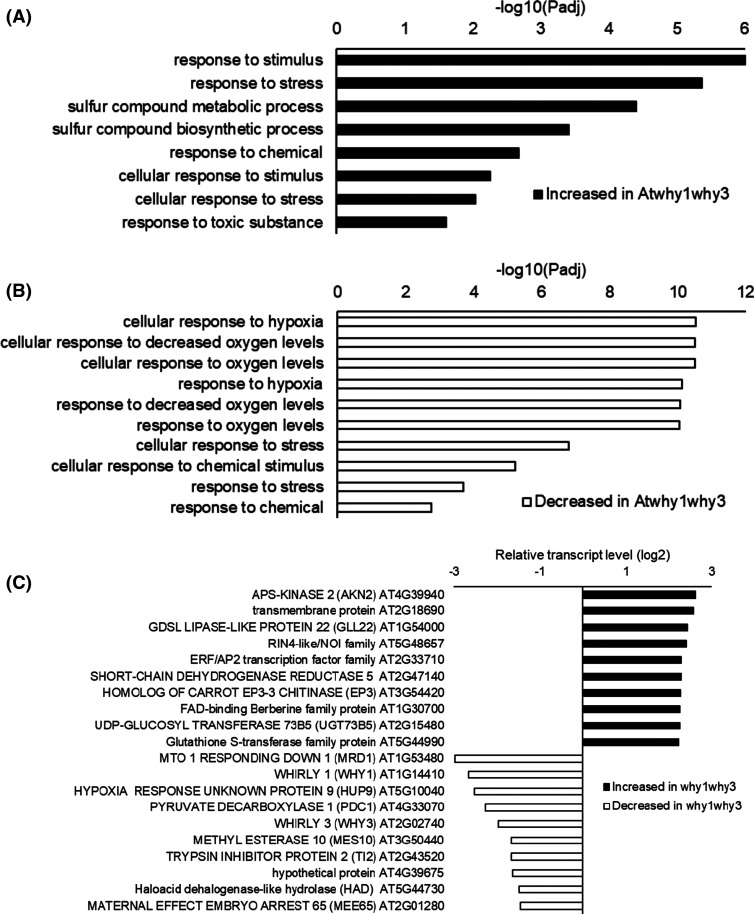
A comparison of the transcript profiles of unaged *Atwhy1why3* mutant seeds compared with the wild type after 6 h of imbibition. Gene ontology (GO) enrichment groups that were decreased in abundance in the imbibed seeds of the *Atwhy1why3* mutants relative to the wild type (**a**), fold change in expression of the 10 most differentially expressed genes (DEGs) showing increased or decreased abundance in the *Atwhy1why3* mutants compared with the wild type (**b**).

A comparison of the transcriptional profiles of imbibed wild type and *Atwhy1why3* mutants revealed significant differences with 52 genes having higher expression and 18 genes with lower transcript levels in the mutants compared the wild type (Figure 8; Supplementary Table S1). Hypoxia-related and oxygen metabolism genes were significantly decreased in the unaged imbibed *Atwhy1why3* mutant seeds relative to the wild type (Figure 8a), with a greater abundance of transcripts encoding stress-related responses. Of the 18 genes with reduced abundance in imbibed *Atwhy1why3* mutant seeds, 50% have hypoxia-related GO annotations and four of these appear in the list of most responsive genes (Figure 8b). The highly significant enrichment of this GO term and is reflected in the *P*-values. Sulfur metabolism-related genes were also more highly expressed in the unaged imbibed *Atwhy1why3* mutants compared with the wild type (Figure 8a). Other genes with high levels of expression in the unaged imbibed seeds of the *Atwhy1why3* mutants compared with the wild type were generally related to the regulation of metabolism (Figure 8b). Transcripts encoding pyruvate decarboxylase (PDC)1, which are key to the establishment of the fermentative metabolism in plants during oxygen shortage were significantly lower in the unaged imbibed seeds of the *Atwhy1why3* mutants compared with the wild type (Figure 8b). Transcripts encoding MATERNAL EFFECT EMBRYO ARREST65 (MEE65) that controls cell proliferation and maternally regulates seed size were also lower in the *Atwhy1why3* mutants than the wild type. Similarly, a small number of transcription factors were differentially expressed at the imbibition stage in unaged *Atwhy1why3* mutant seeds compared with the wild type (Supplementary Figure S3). Of these, the levels of the transcripts encoding the APETALA 2/ethylene response factor (ERF 112) and OXIDATIVE STRESS 2 (OXS2) transcription factors were significantly higher in the *Atwhy1why3* mutant seeds than the wild type (Supplementary Figure S3). Relatively few genes encoding phytohormone-related proteins were differentially expressed at the imbibition stage in unaged *Atwhy1why3* mutant seeds compared with the wild type (Supplementary Figure S3). The seeds were imbibed under optimum conditions and so large changes in stress-related genes may not be expected. Apart from AP2, relatively few genes associated with ethylene signalling were differentially expressed in the imbibed *Atwhy1why3* mutant seeds compared with the imbibed wild type seeds (Supplementary Figure S4). Similarly, only small changes in the levels of transcripts associated with ABA, SA, jasmonate or auxin signalling pathways were detected in the imbibed *Atwhy1why3* mutant seeds compared with the imbibed wild type seeds

#### Imbibed aged seeds

**Figure 9.**
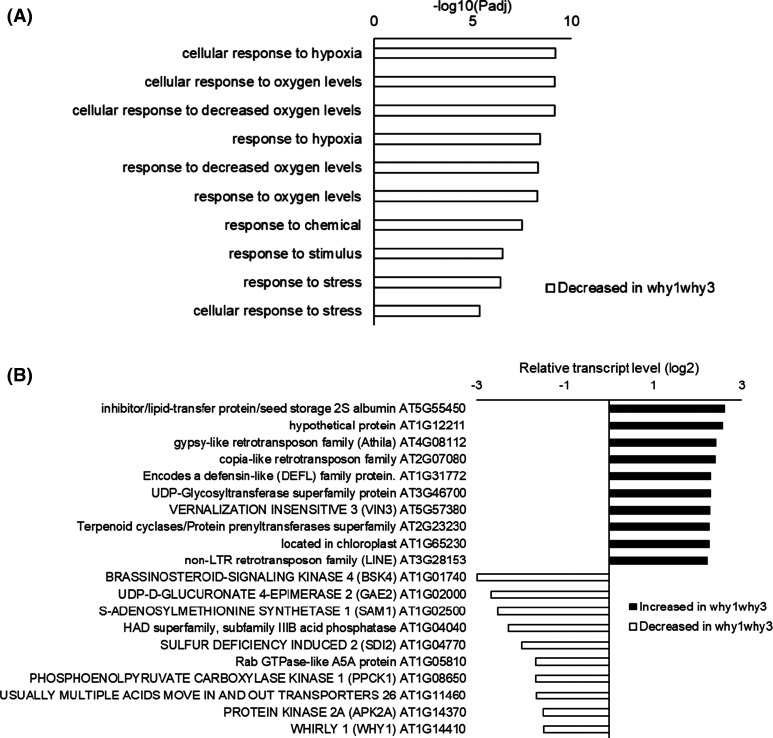
A comparison of the transcript profiles of aged *Atwhy1why3* mutant seeds compared with the wild type after 6 h of imbibition. Gene ontology (GO) enrichment groups that were decreased in abundance in the aged seeds of the *Atwhy1why3* mutants relative to the wild type (**a**) and fold change in expression of the 10 most differentially expressed genes (DEGs) with increased and decreased abundance in the *Atwhy1why3* mutants relative to the wild type (**b**).

The aging treatment resulted in the differential expression of sub-sets of genes the *Atwhy1why3* mutant seeds compared with the wild type (Figure 9). The levels of 118 transcripts were higher while that of 236 transcripts were lower in the *Atwhy1why3* mutant seeds, respectively. Hypoxia-related and oxygen metabolism genes were significantly decreased in the aged *Atwhy1why3* mutant seeds compared with the wild type (Figure 9a). A subset of transcription factors was differentially expressed at the imbibition stage in the aged *Atwhy1why3* mutant seeds compared with the wild type (Supplementary Figure S3). Of these, the levels of transcripts encoding a plant homeodomain protein VERNALISATION INSENSITIVE 3 (VIN3; AT5G57380), which plays a key role in vernalisation responses and in hypoxia tolerance through effects on chromatin remodelling, were higher in the aged *Atwhy1why3* mutant seeds than the wild type (Figure 9b; Supplementary Figure S3). With the exception of transcripts encoding proteins associated with auxin responses, few transcripts encoding phytohormone-related proteins were differentially expressed in the imbibed aged *Atwhy1why3* mutant seeds compared with the imbibed aged wild type seeds than in the unaged controls (Supplementary Figure S4).

## Discussion

Seedling establishment is the first critical step of crop production [3]. While our understanding of the factors that regulate germination has greatly increased in recent years, the molecular mechanisms that control seed vigour and therefore performance, remain poorly understood. The WHY proteins serve important functions in the control of plant development through interactions with key transcription factors [16]. The roles of WHY1 and 3 proteins are demonstrated by the altered silique and seed production observed in the *Atwhy*1, *Atwhy3* and *Atwhy1why3* mutants relative to the wild type controls. In particular, the *Atwhy3* and *Atwhy1why3* mutants had significantly more siliques per plant than the wild type, but with fewer seeds per silique (Figure 4). An earlier study of the *Atwhy*1, *Atwhy3* and *Atwhy1why3* mutants reported that the single mutants had no apparent phenotype relative to the wild type controls [7]. However, a small percentage (less than 5%) of the double knockout plants showed variegation on the leaves, a finding that the authors linked to impaired chloroplast development and function [7]. We did not observe any variegated leaves in the *Atwhy1why3* double mutants in our studies. The differences in the *Atwhy*1, *Atwhy3* and *Atwhy1why3* phenotypes reported here compared with those reported previously are likely due to variations in the growth conditions. However, little information about the growth environment is given in the earlier paper [7], so no firm conclusions regarding phenotypic differences can be drawn.

The Arabidopsis *why2* mutants were found to have lower seed viability [22]. However, no significant differences in germination between the *Atwhy1*, *Atwhy3*, *Atwhy1why3* mutants and the wild type was observed in the absence of accelerated aging in the present study. The data presented here show that the seeds of the *Atwhy1why3* mutants were highly sensitive to accelerated aging. Seed longevity is a complex trait that is regulated by the interaction between environmental and genetic factors, such as GA and ABA, metabolites and antioxidants, cell maintenance factors, and seed coat composition [2,28,29]. The transcriptome profiles of the imbibed and aged seeds reported here reveals that the expression of oxygen availability and hypoxia-related genes correlated with the success of seed germination.

Transcriptome profiling approaches have been widely used to analyse the molecular basis for plant development [30]. A previous analysis of the imbibition-induced changes in the seed transcriptome profile [26] revealed that the turnover of maturation transcripts was largely complete following 6 h imbibition, with increases in transcripts encoding metabolic, transcription, protein synthesis and cell cycle related components increasing by 12 h imbibition. While global patterns of gene expression were altered in pea seeds in response to progressive accelerated aging [31], there are few reports of the transcript changes occurring during the aging of Arabidopsis seeds [32] The present study revealed that there was little overlap in the patterns of gene expression observed in response to imbibition in the aged and unaged seeds. Hence the imbibition programme was disrupted as a result of seed aging. The seed coat creates a dry, low oxygen environment in dry seeds that is alleviated upon imbibition [27]. The greatest imbibition-induced increases in gene expression observed in the wild type seeds (Figure 6) encode proteins involved in oxygen sensing and responses to hypoxia. Conversely, GO terms associated with water deprivation, dehydration responses and ABA were decreased following imbibition. Together, these findings demonstrate that germination requires a strong response to the low oxygen environment of the seed at the early stages of germination. Although there were no significant differences in the germination of the mutants and the wild type (in high-quality seeds), the aged *Atwhy1why3* seeds showed decreased expression of hypoxia-related and oxygen metabolism genes relative to the wild type (Figure 8). These findings suggest that imbibition-induced relief of hypoxia was impaired in the aged *Atwhy1why3* seeds and that this prevented germination. Thus, the WHY1 and WHY3 proteins function in regulating the hypoxia responses of seeds. DNA and RNA integrity can be compromised during seed aging, in particular as seed vigour declines towards the threshold of viability loss. However, no changes in the expression of genes encoding proteins involved in DNA damage repair were detected in aged *Atwhy1why3* seeds.

Transcripts encoding seed storage-related proteins that function in protection during germination and seedling formation [33] were generally much more abundant in high-quality seeds of the wild type than those of the *Atwhy1why3* mutants. Several transcripts (*AT1G27360*, *AT1G27370*, *AT1G69170* and *AT5G43270*) encoding microRNAs such as miR156 and miR157, which are Squamosa-promoter Binding Protein (SBP)-like proteins [34,35] were altered in abundance in the *Atwhy1why3* mutant seed relative to the wild type. Changes in the expression of sub-sets of transcription factors were observed at the imbibition stage in unaged *Atwhy1why3* mutant seeds compared with the wild type. The levels of transcripts encoding APETALA 2/ethylene response and OXIDATIVE STRESS 2 transcription factors are significantly higher at the imbibition stage in the unaged *Atwhy1why3* mutant seeds than the wild type but not in the imbibed aged seeds. These proteins may be linked in the regulation of seed germination through effects on ABA signalling [37,38]. A recent analysis of seedling development in Arabidopsis ABA hypersensitive mutants reported that OXIDATIVE STRESS 3 (OXS3) is a negative regulator of ABI4 expression through interactions with AFP1 (ABI FIVE BINDING PROTEIN 1) and histone H2A.X [39]. Although the functions of the OXIDATIVE STRESS 2 are unknown but it is possible that this transcription factor also regulates ABA signalling during germination, especially as WHY protein occupancy alters histone lysine modification in a developmental manner in Arabidopsis [39].

The large number of transcripts encoding HSPs that appear in seeds after aging suggests that these proteins have physiological importance [38–40]. Differences in the levels of several transcripts encoding HSPs was observed between the *Atwhy1why3* mutant seeds compared with those of the wild type. For example, transcripts encoding HSP70 were generally abundant in unaged wild type seeds but not in those of the *Atwhy1why3* mutants. The induction of HSPs and sHSPs in late seed maturation has previously been associated with a protective role in seed longevity [39]. While small heat HSPs are also important in seed development and embryo maturation [39], little information is available on the role of HSPs in seed aging. Transcripts encoding the rice HSP called OsHSP18.2 were significantly increased during seed aging and found to be important in seed germination, vigour and seedling establishment under stress [40]. Overexpression of WHY1 in tomato led to increased levels of SlHSP21.5A transcripts and enhanced protection against heat stress [41]. Furthermore, overexpression of the sunflower HaHSFA9 heat stress transcription factor in transgenic *Nicotiana tabacum* seeds increased resistance to the adverse effects of accelerated aging [42]. Like HSPs, LEA proteins are important in seed desiccation tolerance and longevity [40]. LEA proteins accumulate during seed maturation and in response to abiotic stresses as well as dehydration [39]. Both dehydrins and LEA proteins have roles in seed longevity during the dry dormant stage, as well as in germination and in responses to salinity [43]. A decrease in tomato seed storability was observed after priming can be mitigated by heat shock post-priming [43–45].

Previous studies have found that *Atwhy1* mutants had reduced sensitivity to ABA and SA, whilst transgenic lines overexpressing AtWHY1 were found to be hypersensitive to ABA and overexpressing only the nuclear form of AtWHY1 led to the same level of ABA insensitivity as *Atwhy1* mutants [15]. However, the transcript profiling analysis reported here does not provide any further insights into the role of the WHY proteins in the SA or ABA-mediated controls of germination.

The most novel and interesting finding reported here is the requirement for the WHY proteins in oxygen sensing and potentially regulation of the N-degron pathway. Plants have an oxygen-sensing system that involves the O_2_-dependent destruction of protein substrates with amino-terminal Cys groups through the plant cysteine oxidase (PCO) branch of the PROTEOLYSIS (PRT)6 N-degron pathway [46,47]. This branch of the N-degron pathway controls the proteolysis of transcriptional regulators of plant growth and development in relation to oxygen availability. The group VII ETHYLENE RESPONSE FACTOR (ERFVII) transcription factors are major substrates of the pathway, integrating oxygen availability to development during seedling establishment [46]. Moreover, mitochondrial retrograde signalling represses the PCO-dependent N-degron pathway [48]. Under the low-oxygen conditions that occur in dry and imbibed seeds, ERFVIIs are stabilised due to inhibition of PCO activity, resulting in the induction of hypoxia-related gene expression [49], as observed in the imbibed high-quality (unaged) seeds. Of the genes with lower abundance in imbibed *Atwhy1why3* mutant seeds, 50% have hypoxia-related GO annotations. WHY1/3-dependent regulation of hypoxia-related gene expression is therefore an important component regulating oxygen signalling during seed imbibition. The levels of PDC1 transcripts were significantly lower in the imbibed *Atwhy1why3* mutant seeds than the wild type. PDC is a mitochondrial enzyme that is required for hypoxia tolerance by regulating the supply of energy through fermentative metabolism and the pyruvate dehydrogenase by-pass, through which pyruvate is converted to acetyl-CoA [50,51]. Taken together, these findings show that oxygen sensing is impaired when the *Atwhy1why3* mutant seeds are aged and hence germination is impaired.

In summary, the data presented here demonstrates a strong link between the WHY1 and three proteins and susceptibility to seed aging. The findings reported here also provide new insights into factors that might serve to better protect seeds and preserve germination. The WHY1 and three proteins are therefore attractive targets for increasing seed vigour and viability. There is considerable potential for translation of this knowledge to improve sustainable agriculture, since manipulation of *WHY* gene expression may result in seeds that are better able to withstand aging.

## Material and methods

### Plant material

The *Arabidopsis thaliana* Col-0 wild type (WILD TYPE) was compared with *Atwhy* mutants (*Atwhy1*, *Atwhy3*, *Atwhy1why3*) in the following studies. The *Atwhy1* with a TDNA in the promoter at nucleotide −102 relative to the initial ATG which originated from the Salk Institute Genomic Analysis Laboratory (La Jolla, CA, U.S.A.) and the *Atwhy3* line is a TILLING line with a mutation that changes the TGG codon at position W138 to a TGA stop codon which is from the Seattle TILLING Project [21]. The *Atwhy1why3* double mutant was the crossed progeny of the two single mutants and was provided by the Normand Brisson group (Department of Biochemistry, Université de Montréal, Montréal, Canada).

### Seed aging assays

Aliquots of each of the genotypes were sealed in an air-tight container at 35°C and 83% relative humidity (maintained by a saturated KCl solution) for 7 days or 14 days.

### Analysis of seed viability and vigour

Seeds were plated on blue blotter germination paper with 7 ml of dH_2_O and cold stratified at 4°C for 48 h to remove dormancy. They were placed in a 20°C growth chamber with 60% RH and a 16 h photoperiod (65–95 µmol m^−2^ s^−1^ irradiance). Seeds were scored for germination by the emergence of the radicle from the endosperm. After 8 days the number of germinated seeds was counted and recorded. The half inhibitory time (ID_50_) refers to the time required for the seed germination rate to reduce to half the germination rate of the high-quality seeds [23]. A large ID_50_ value shows slower decreases of seed germination with aging which means higher longevity of seeds.

### RNA-Seq analysis

Wild type and *Atwhy1why3* mutants were analysed by RNA-seq. Seed treatments were as follows: unaged seeds with 0 h imbibition, unaged seeds with 6 h imbibition, aged 7-day seeds with 6 h imbibition. The Promega SV Total RNA Isolation System kit (Madison, WI, U.S.A.) was used to extract total RNA according to the manufacturer's instructions [24]. Spectrophotometric quantification at 260 nm and samples between 1.7–2.1 A260/S280 ratio was accepted as sufficient purity (Nanodrop spectrophotometer). Total RNA samples were analysed by paired end reads using the Illumina NovaSeq 6000 Sequencing System (Novogene, Cambridge, U.K.). The paired and unpaired read data which was analysed using the Galaxy software (UseGalaxy.eu), produced by the Freiburg Galaxy Team [25]. Quality control was performed at each stage using FASTQC and MULTIQC. FASTQ files were filtered using trimomatic and aligned to the TAIR10 Arabidopsis genome using HISAT2. Paired and unpaired alignments were combined using samtools merge and featurecounts using to quantify gene fragments. Deseq2 was used to calculate normalised gene expression and identify differentially expressed genes (Supplementary Table S1), filtering for FDR values <0.01 and >2-fold changes in expression. Gene ontology enrichment was analysed using g:Profiler (https://biit.cs.ut.ee/gprofiler/gost).

### Plant growth conditions

Plants of each genotype were grown in a controlled environment chamber with a 20°C/16°C day/ night temperature regime in a 16 h photoperiod (65–95 µmol m^−2^ s^−1^ irradiance) and 60% relative humidity in pots of compost (Sinclair Professional potting compost, Sinclair Pro, Cheshire) on p15 cell trays.

### Plant biomass

Soil was washed from the plants and weighed. Afterwards, these plants were placed into Glassine bags and sealed inside a 100°C oven for 48 h then weighed.

### Seed numbers and silique yield

The number of siliques were counted on each branch of each plant. Additionally, on each plant three random siliques were removed carefully and opened to count seeds per silique.

## Data Availability

All primary data and datasets are available from the authors upon request. The RNA-seq data are available in the NCBI Sequence Read Archive under BioProject ID PRJNA918543 (https://www.ncbi.nlm.nih.gov/bioproject/918543.

## Open Access

Open access for this article was enabled by the participation of University of Birmingham in an all-inclusive *Read & Publish* agreement with Portland Press and the Biochemical Society under a transformative agreement with JISC.

## Abbreviations

ABA: abscisic acid
GA: gibberellic acid
HSPs: heat shock proteins
PCO: plant cysteine oxidase
PDC: pyruvate decarboxylase
SA: salicylic acid
WHY: WHIRLY
