# Supplementary material for: WHIRLY proteins maintain seed longevity by effects on seed oxygen signalling during imbibition
Source: Biochem J. 2023 Jul 6;480(13):941–56. doi: 10.1042/BCJ20230008 (PMC10422932; doi:10.1042/BCJ20230008)
Supplement: Supplementary Material 1 [file BCJ-480-941-s1.pdf]

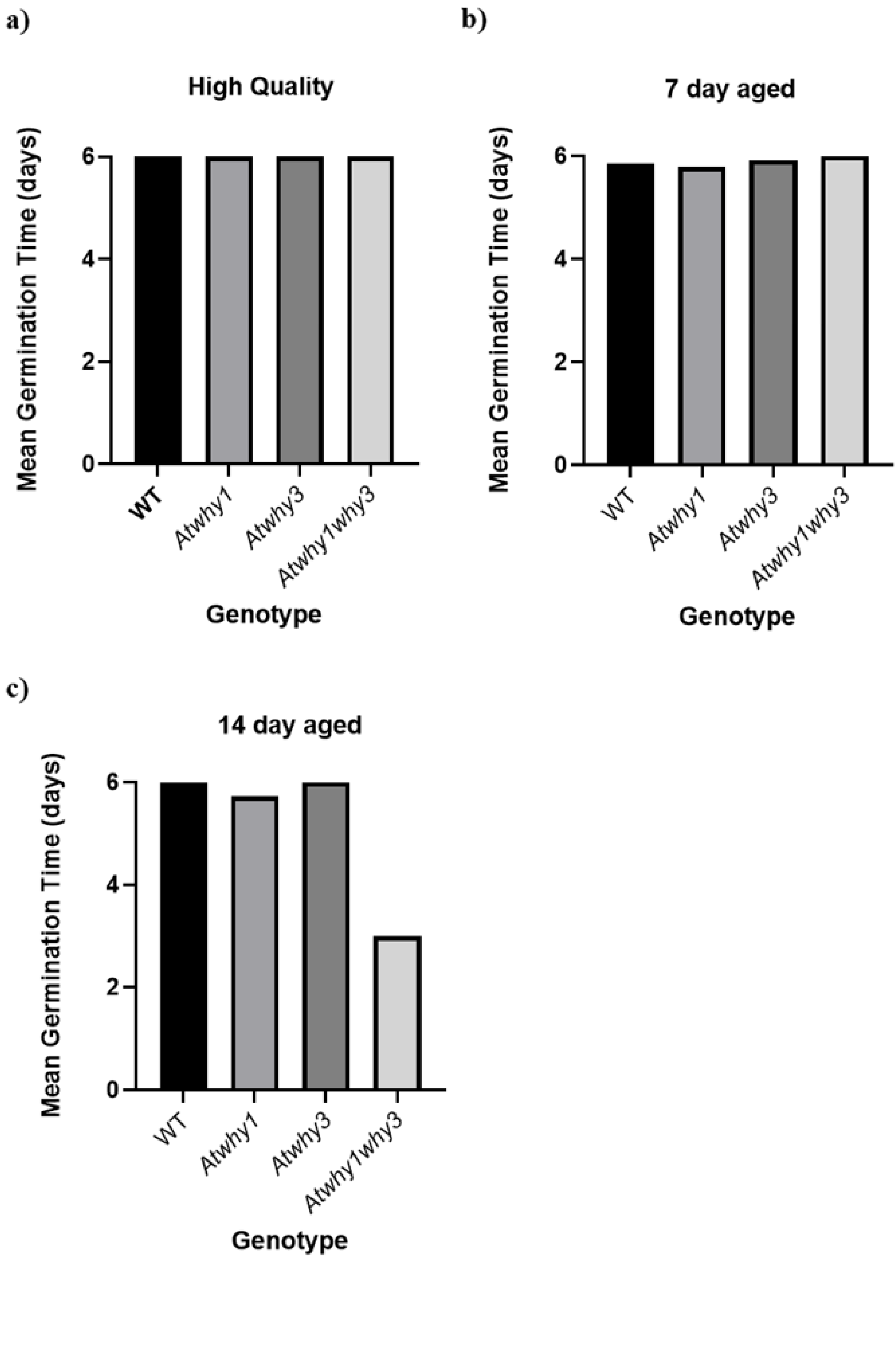


Supplementary Figure 1: The mean germination time (Σ(nxd) /N) of the three *Atwhy* mutant seeds compared to the WT at 7 days after cold stratification in a) high quality unaged seeds; b) 7 day aged seeds; and c) 14 day aged seeds. Each treatment is shown per genotype.

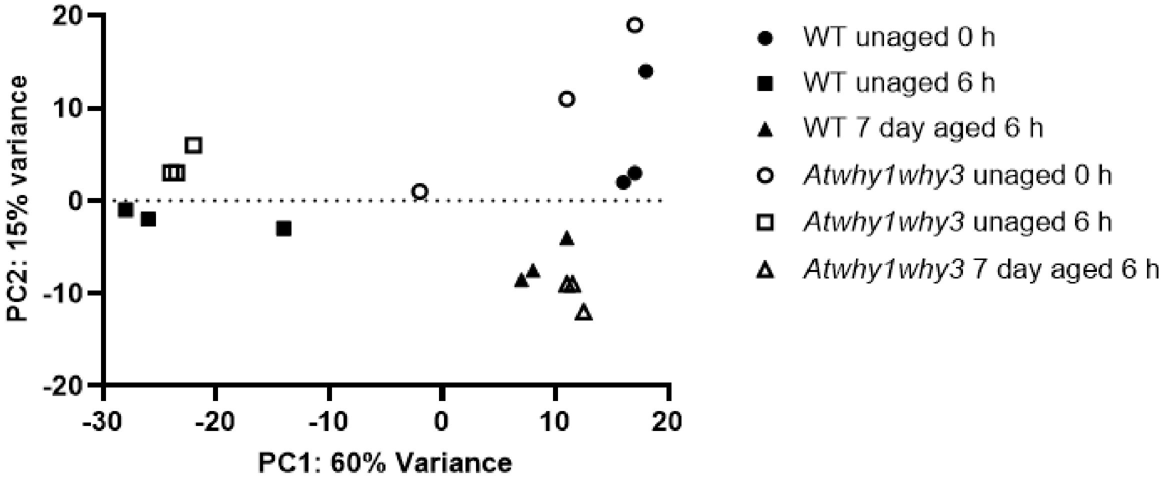


Supplementary Figure 2: Principal component analysis (PCA) biplot of 18 samples of RNA-seq data.

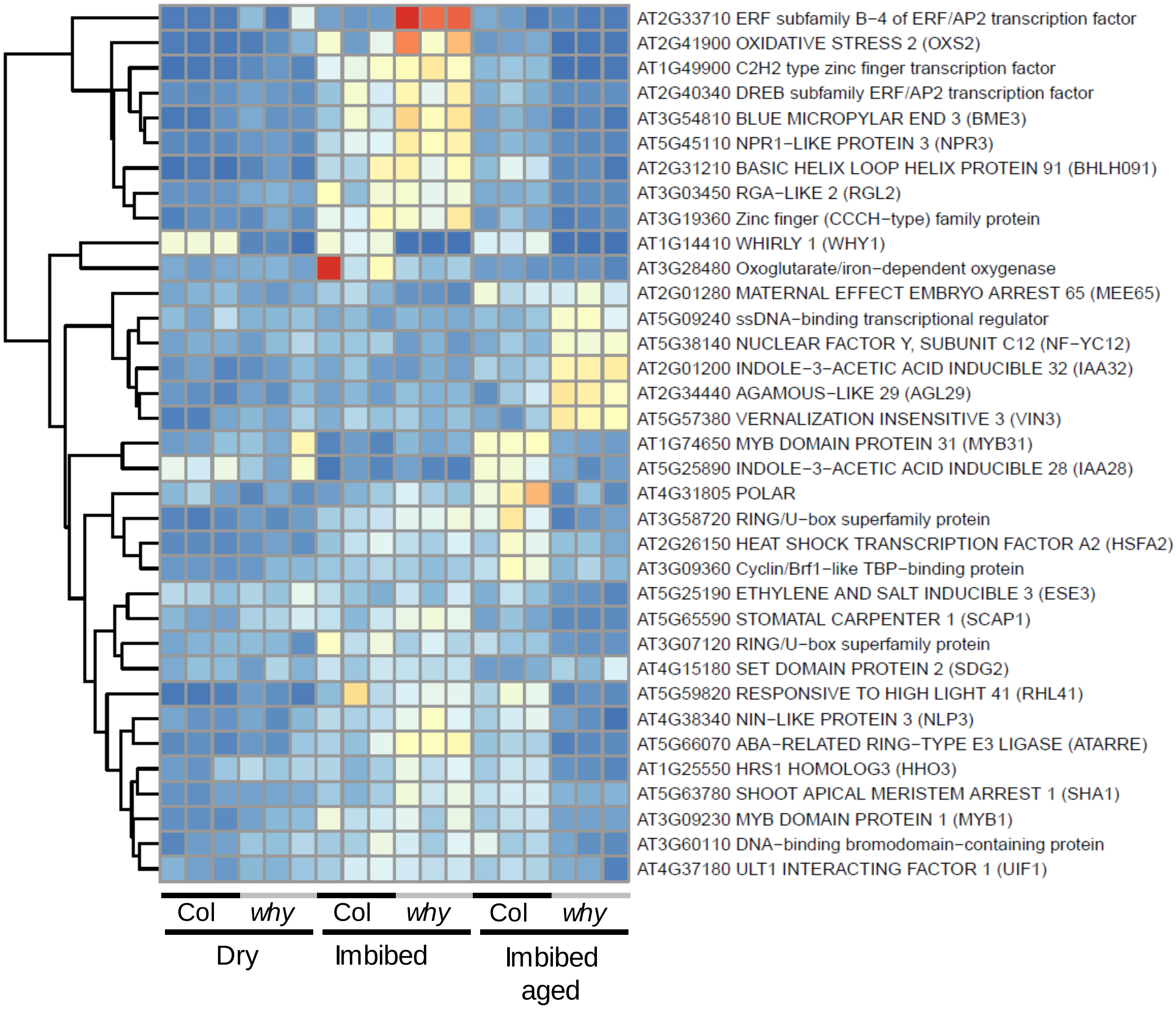


Supplementary Figure 3. Differentially expressed transcription factors in pairwise comparisons of wild type and why1why3 mutant imbibed or aged imbibed seeds. Transcription factors were provided by the Jen Sheen lab https://molbio.mgh.harvard.edu/sheenweb/AraTRs.html

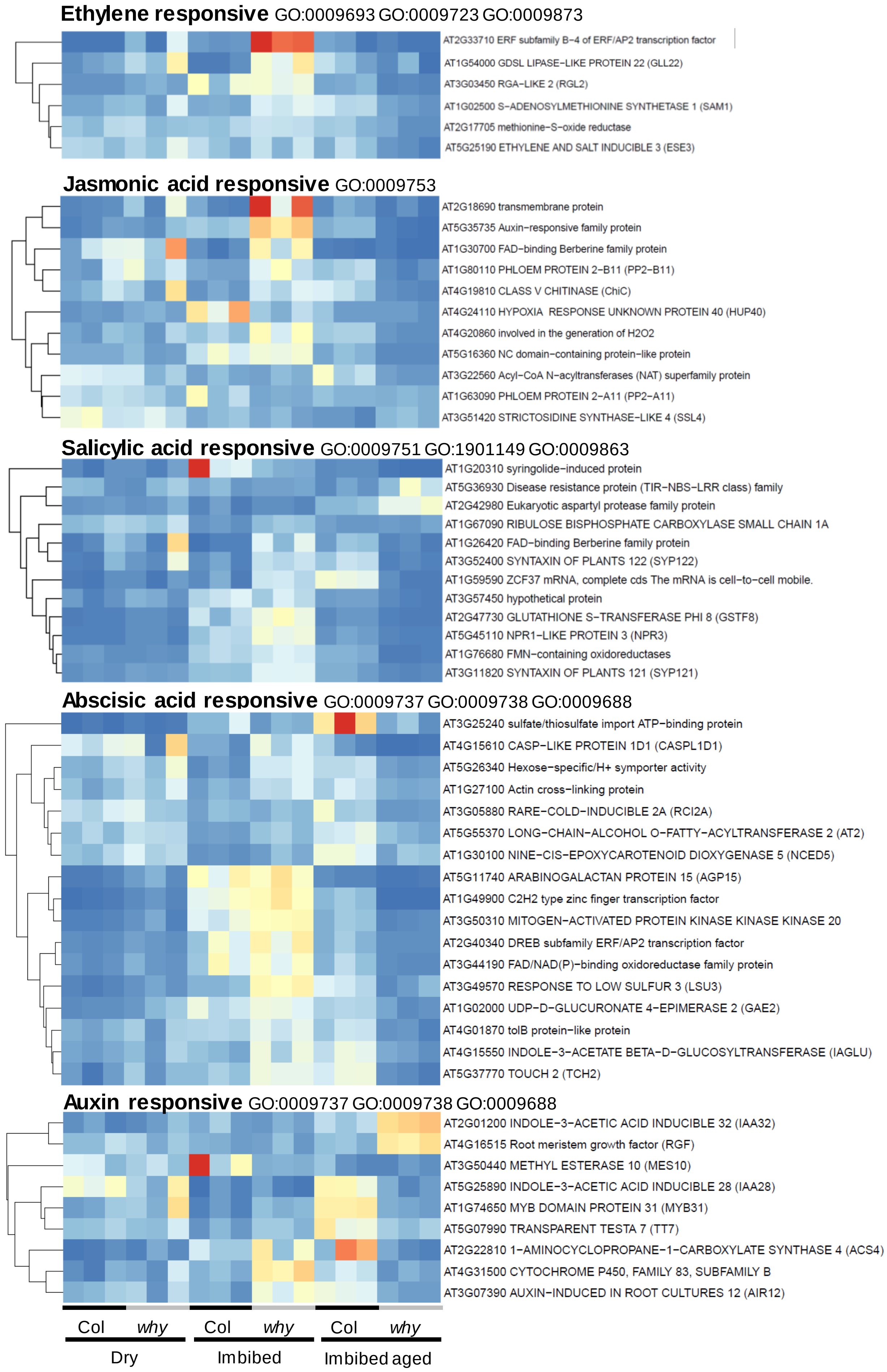


Supplementary Figure 4. Differentially expressed hormone responsive factors in pairwise comparisons of wild type and why1why3 mutant imbibed or aged imbibed seeds. Hormone responsive transcripts were identified using the gene ontology (GO) terms indicated.
